# Landscape variability explains spatial pattern of population structure of northern pike (*Esox lucius*) in a large fluvial system

**DOI:** 10.1002/ece3.1121

**Published:** 2014-09-03

**Authors:** Geneviève Ouellet-Cauchon, Marc Mingelbier, Frédéric Lecomte, Louis Bernatchez

**Affiliations:** 1 Université Laval, Institut de Biologie Intégrative et des Systèmes (IBIS)1030 Avenue de la Médecine, Québec, Québec, G1V 0A6, Canada; 2 Ministère du Développement durable, de l'Environnement, de la Faune et des Parcs du Québec (MDDEFP), Service de la Faune Aquatique880 chemin Sainte-Foy, Québec, Québec, G1S 4X4, Canada

**Keywords:** Conservation, environmental variation, *Esox lucius*, habitat variability, landscape genetics, management, population genetic structure

## Abstract

A growing number of studies have been investigating the influence of contemporary environmental factors on population genetic structure, but few have addressed the issue of spatial patterns in the variable intensity of factors influencing the extent of population structure, and particularly so in aquatic ecosystems. In this study, we document the landscape genetics of northern pike (*Esox lucius*), based on the analysis of nearly 3000 individuals from 40 sampling sites using 22 microsatellites along the Lake Ontario – St. Lawrence River system (750 km) that locally presents diverse degrees of interannual water level variation. Genetic structure was globally very weak (*F*_ST_ = 0.0208) but spatially variable with mean level of differentiation in the upstream section of the studied area being threefold higher (*F*_ST_ = 0.0297) than observed in the downstream sector (*F*_ST_ = 0.0100). Beside interannual water level fluctuation, 19 additional variables were considered and a multiple regression on distance matrices model (*R*^2^* *=* *0.6397, *P *<* *0.001) revealed that water masses (*b *=* *0.3617, *P *<* *0.001) and man-made dams (*b *=* *0.4852, *P *<* *0.005) reduced genetic connectivity. Local level of interannual water level stability was positively associated to the extent of genetic differentiation (*b *=* *0.3499, *P *<* *0.05). As water level variation impacts on yearly quality and localization of spawning habitats, our study illustrates how temporal variation in local habitat availability, caused by interannual water level fluctuations, may locally decrease population genetic structure by forcing fish to move over longer distances to find suitable habitat. This study thus represents one of the rare examples of how environmental fluctuations may influence spatial variation in the extent of population genetic structure within a given species.

## Introduction

Landscape genetic studies have mostly focused on long-term processes that structure populations (Manel and Holderegger 2013), although a growing number of studies have been investigating the influence of contemporary environmental factors on population genetic structure. However, few have addressed the issue of spatial patterns in the variable intensity of factors, such as short-term (e.g., interannual) fluctuations, influencing the extent of population structure. Habitat variability influences various aspects of population dynamics [e.g., population size and persistence (Nelson et al. 1987), reproductive success (Titus and Mosegaard 1992) and dispersal (Messier et al. 2012)]. Therefore, its impact should be integrated in conservation and management strategies (Ostergaard et al. 2003). Temporal landscape variation has also been shown to affect the temporal stability of population genetic structure in insects (Baggiano et al. 2011), amphibians (Fitzpatrick et al. 2009), mammals (Messier et al. 2012), and freshwater fishes (Ostergaard et al. 2003). In those studies, genetic structure was mostly assessed in a uniformly unstable habitat, but, to our knowledge, no study has investigated the consequences on population genetic structure of different levels of habitat variability compared to other environmental factors.

**Figure 1:**
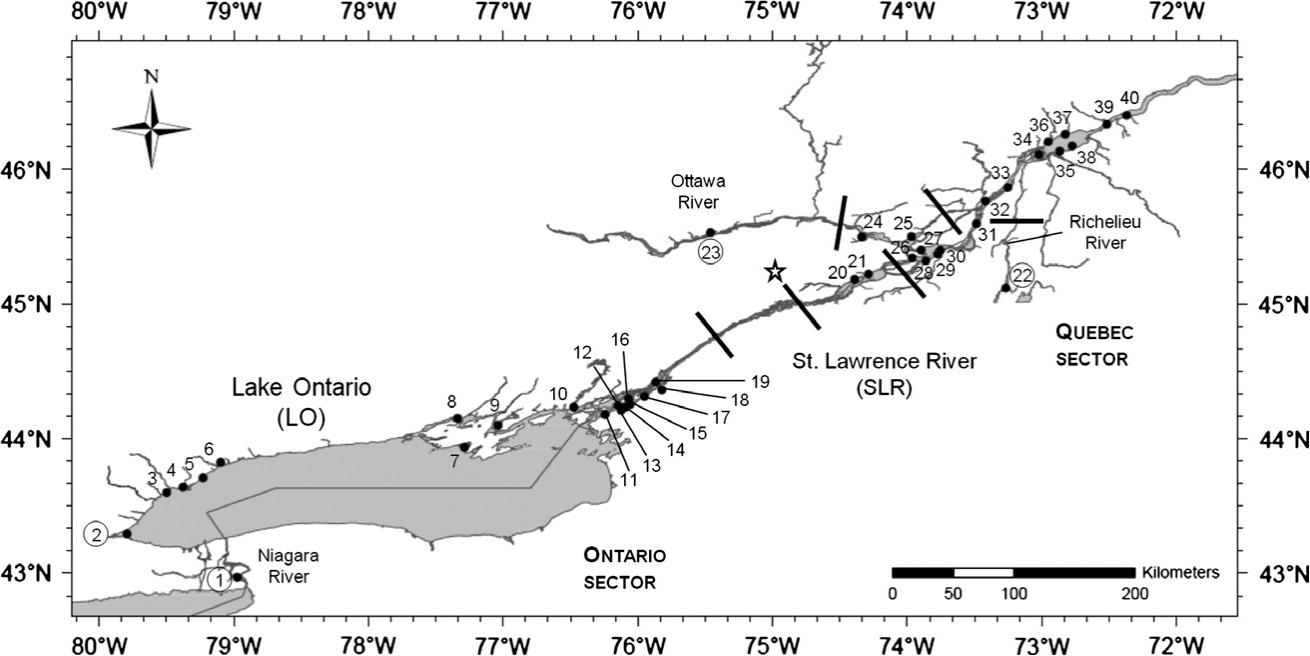
Sampling sites in LO–SLR and their tributaries. Annexes are circled. Lines: localization of dams. Star: Moses-Saunders regulation dam.

To this end, the Lake Ontario – St. Lawrence River system from the Great Lakes basin in North Eastern North America represents a particularly relevant study area (Fig.1). More specifically, Lake Ontario (LO), being 26,435 km^2^, is limited downstream by the Moses-Saunders dam (Fig.1) and located at the head of several important rapids. LO discharges into the St. Lawrence River (SLR) that is a large and complex river stretch comprising three large fluvial lakes separated by rapids and narrow corridors (from West to East, Lake St. Francis, Lake St. Louis and Lake St. Pierre). Since the 1850s, the SLR experienced major transformations, including dredging of a large navigation channel and building of several important dams between 1914 and 1980 (Fig.1).

There are numerous tributaries flowing in the SLR, among which the largest are the Ottawa River and Richelieu River. The discharges of those tributaries, which have greatly distinctive physical and chemical characteristics (Table S1), are flowing side by side in SLR corridor with very limited mixing area so that those water masses lead to very contrasting fish habitat conditions (CSL 1996). Moreover, the SLR tributaries have important effects on fish habitat. To begin with, varying climatic conditions largely increase seasonal variations in water discharge. By driving water flow fluctuations in the system, this naturally cause interannual water level variations, leading to higher water level fluctuations throughout the year and between years within the SLR than in LO. Moses-Saunders dam was built in 1960 in the upstream part of SLR corridor (Fig.1) to seasonally and interannually control water level, a role supplemented by other hydroelectric dams. Nevertheless, water levels of the “Ontario sector,” comprising LO and upper SLR, remain considerably stable on an interannual basis, in contrast to downstream dam complex in the “Quebec sector,” represented by the lower SLR, where water level increasingly interannually fluctuates toward downstream. Interannual water level variation coefficient is ˜0.4 m in LO compared with ˜13 m in Lake Saint-Pierre (Ministère du Développement Durable, de l'Environnement, de la Faune et des Parcs du Québec, unpubl. data). Such water levels variation considerably shape fish spawning habitat by influencing local water depth that in turn influences vegetation localization and density, water temperature and water velocity (Mingelbier et al. 2008). Furthermore, for fishes spawning in floodplain, water level variation influences young-of-the-year survival and year-class strength since hasty spring water retrieval can cause massive mortality of eggs and larvae (Mingelbier et al. 2008).

Northern pike (*Esox lucius*), one of the most widely distributed freshwater species in the northern hemisphere, is one of the most widely distributed in this system and largely influenced by the amount of shallow habitats at different stages of its life history. It is mainly sedentary (Bosworth and Farrell 2006; Vehanen et al. 2006) but there is also some evidence of a certain level of natal-site spawning fidelity (Miller et al. 2001), although philopatry rate remains undefined. In the LO–SLR system, northern pike spawns in early spring during the flood in shallow and lentic warmer waters of wetlands where dense vegetation occurs (Casselman and Lewis 1996; Farrell 2001) and thus could be extensively influenced by water level variation (Mingelbier et al. 2008).

Our general objective was to investigate the possible consequences of the extent of habitat short-term fluctuations in spatial availability on the population genetic structure pattern of northern pike in relation to other landscape features that could putatively affect connectivity in the system. We thus documented the number of genetically distinct populations in the system, as well as their spatial distribution and level of connectivity. We then assessed which environmental factors best explain the observed pattern of population genetic structure by means of a landscape genetics approach. To our knowledge, this study represents one of the rare landscape genetic studies performed on such a large-scale freshwater landscape and the first to assess the consequences of spatial variation in the extent of short-term fluctuations in habitat availability.

## Material and Methods

### Sample collection

Between 20 and 162 individuals were sampled at 40 sites in 2007, 2009, and 2010 (Fig.1 and Table S2), totaling 2913 pikes. The 750-km long study area was subdivided in two regions based on the occurrence of the delineation between LO and SLR (Moses-Saunders dam and rapids) and because of the lack of samples over 150 km in the middle due to lack of suitable northern pike habitat: (1) upstream “Ontario sector” including LO and upper SLR and (2) downstream “Quebec sector” comprising lower SLR. Four sampling sites were geographically isolated from the main study system as they were either tributaries or isolated basins and hence here they are subsequently named “annexes” (sites 1-2-22-23; Fig.1 and Table S2). Fin clips sampling mainly occurred during the spawning period (March 15^th^ to May 31^st^; Farrell et al. 2006), although several samples were collected slightly before or after spawning (Table S2). Before spawning time, angled northern pikes were sampled during the ice fishing season and, during and after spawning, trap nets were used.

### Genetic data

Genomic DNA was extracted from fins clips preserved in 95% ethanol using a salt extraction method (Aljanabi and Martinez 1997). Twenty-two microsatellite markers (Table S3) were amplified with multiplex PCRs using Bio-Rad IQ Supermix. Twelve of these were previously published (Miller and Kapuscinski 1997; Genetic Identification Service Inc unpublished data; Aguilar et al. 2005), whereas we developed the other 10 markers (Ouellet-Cauchon et al. 2014). PCR program was: 3 min at 95°C, [30 sec at 95°C, 30 sec at 56°C or 60°C, 30 sec at 72°C]_35_, 30 min at 60°C. Electrophoresis of the amplified loci was completed using an Applied Biosystems 3130xl Genetic Analyzer (Life Technologies, Waltham, MA)and the raw data were treated with DATA COLLECTION v3.1.1. (Life Technologies) Genotypes were resolved using GENEMAPPER v.4.1 (Life Technologies).

### Intrapopulation diversity and structure

*F*_*is*_ values and exact tests for deviations from Hardy–Weinberg proportions and linkage equilibrium were computed using GENEPOP (Raymond and Rousset 1995) with Bonferroni corrections (*α *= 0.05; Rice 1989). We evaluated expected (*H*_*E*_) and observed (*H*_*O*_) heterozygosity with GENETIX v.4.05.2 (Belkhir et al. 2004), allelic richness (*Ar*) and private allelic richness (*Apr*) over all loci standardized for smallest sample size (*N *=* *20) with HP-RARE (Kalinowski 2005). Temporal stability of genetic structure between 2009 and 2010 samples was assessed with an analysis of molecular variance (AMOVA; ARLEQUIN v.3.1, Excoffier et al. 2005) performed on sites with *N *>* *20 each year (2007 excluded because it had only one sample). We also assessed the stability of genetic structure as a function of sampling period (prior and during spawning period). Given the results of both AMOVAs (see Results section), temporal samples were pooled for further analyses.

### Population structure

Pairwise *F*_ST_ values were computed with GENETIX v.4.05.2 (Belkhir et al. 2004) with a FDR correction of *P *<* *0.05 (Benjamini and Hochberg 1995). Isolation by distance (IBD) was tested with Mantel tests (Mantel 1967) and pairwise *F*_ST_/(1-pairwise *F*_ST_) were plotted as a function of the shortest waterway geographic distance between sites (Rousset 1997). Mantel's tests were computed for the whole study area and for comparisons within the Ontario and within the Quebec sectors excluding the four annexes. Genetic population structure was unsuccessfully assessed with STRUCTURE software (Pritchard et al. 2000), and incongruous and unstable clusters were obtained because of weak global level of genetic differentiation (see Results below; Latch et al. 2006; Jones and Wang 2012). A DAPC analysis (Jombart et al. 2010) revealed no obvious clustering pattern as well (results not shown). Consequently, we used the BARRIER software v.2.2 to identify putative barriers to dispersal between all sampling sites and the 20 first barriers in terms of relative strength were first retained (Manni et al. 2004). Barrier relative strength was evaluated by calculating ratio converted into percentage of mean pairwise *F*_ST_ over all crossed edges from every pair of sites of a given barrier and maximum pairwise *F*_ST_ observed (Manni et al. 2004). Barriers strength was plotted as a function of downstream waterway distance, the starting point being western LO (site 2) and without considering barriers isolating SLR annexes. Barriers boundaries with significant *P*-values were used to delimit population groupings and genetic variance explained by these was quantified using AMOVA (ARLEQUIN v.3.1, Excoffier et al. 2005). Pairwise *F*_ST_ values were also assessed between groupings. To assess the relative extent of genetic structure across the system, global *F*_ST_ values were computed for the whole study area, for the Ontario and Quebec sectors separately, both with and without the four annexes, with GENETIX v.4.05.2 (Belkhir et al. 2004).

### Landscape genetics

Landscape genetic analysis was performed on the Quebec sector only (lower SLR; except for water level fluctuation, see below) as available environmental data were not sufficiently detailed for the LO region, and also because of the large sampling gap of 150 km between the upper and lower SLR, which is an important caveat for landscape genetics analysis (Manel et al. 2003). Pairwise environmental distance matrices for values at sampling sites were built for 20 environmental variables (Table S4). To select variables most explicative of the observed pattern of genetic structuring and to avoid including too many variables in the same multivariate model, Mantel tests were firstly performed (Mantel 1967) for pairwise *F*_ST_ matrix coupled to each pairwise environmental distance matrices. Secondly, Mantel tests were performed to test for collinearity between selected environmental variables (Mantel 1967), which led to removal of the pH variable which was highly correlated with spring water conductivity (Mantel's *r *=* *0.86, *P *<* *0.05). Thirdly, to assess the relative contribution of the retained variables to the model, a multiple regression on distance matrices model (MRM; Legendre et al. 1994) was computed on selected variables with the ecodist R package (R Development Core Team 2012). A full model was run with all six standardized environmental variables, and the following final model only included the significant variables from full model. For each model, Pearson's correlation coefficient and 9999 permutations were computed.

Finally, the relationship of the interannual water level variation with genetic structure was further evaluated over the whole study area. We divided the sampling area in windows of 50 km width with a 25 km sliding. We then computed mean pairwise *F*_ST_ values between sites within each window and mean of interannual water level variation coefficients calculated for each sampling site (Ministère du Développement Durable, de l'Environnement, de la Faune et des Parcs du Québec, unpubl. data) within each window. Mean pairwise *F*_ST_ values and mean interannual water level variation coefficients were plotted as a function of downstream waterway distance (from site 2) for each window. Moreover, mean pairwise *F*_ST_ values were plotted as a function of mean interannual water level variation coefficients for each window for both upstream (Ontario sector) and downstream (Quebec sector) of the Moses-Saunders regulation dam. Spearman correlation coefficients were assessed. When not mentioned otherwise, analyses where computed with R software (R Development Core Team 2012).

## Results

### Intra-population diversity and structure

Compared with other studies conducted in freshwater fishes, genetic diversity was modest and comparable among sampling sites (mean *H*_*E*_ = 0.63 [range = 0.57–0.66], mean *H*_*O*_ = 0.63 [range = 0.54–0.68], mean *Ar* = 5.65 [range = 4.63–5.99], mean *Apr* = 0.05 [range = 0.01–0.16]; Table S2). Mean *F*_IS_ on all loci was low (0.01 [range = −0.05–0.07]; Table S2) and Hardy-Weinberg and linkage disequilibrium tests detected no consistent pattern (Table S2).

### Population structure

The AMOVA between 2009 and 2010 samples revealed variation between years which was 10 times smaller than the extent of spatial genetic structure (0.12% vs. 1.38%, *P *<* *0.01; Table S5A). With a second AMOVA, significant but very weak variation was found between sampling periods relative to spatial structuring (0.13% vs. 1.22%, *P *<* *0.05; Table S5B). Given this, temporal samples were pooled for further analyses.

Given its geographic scale, the global extent of genetic structure across the whole study area (more than 750 km) was weak (global *F*_ST_ = 0.0208, *P *<* *0.001). Pairwise *F*_ST_ values ranged between −0.0037 and 0.1123 (Table S6). The four annexes were the most divergent sites [mean pairwise *F*_ST_ values with all sites: site 1, 0.064; site 2, 0.091; site 22, 0.028; site 23, 0.028]. IBD relationship was significant (Mantel's *r *=* *0.4812, *P *=* *0.0001) but moderate for the whole study area (slope = 6.17 × 10^−5^, *P *<* *0.00001). However, genetic divergence increased more steeply with waterway geographic distance within the Ontario sector (slope = 6.32 × 10^−5^, *P *<* *0.00001) than within the Quebec sector (slope = 2.06 × 10^−5^, *P *=* *0.00308) and waterway distance better explained the genetic distance within the former sector (Mantel's *r *=* *0.4905, *P *=* *0.0001) than the latter sector (Mantel's *r *=* *0.2454, *P *=* *0.0038).

**Figure 2:**
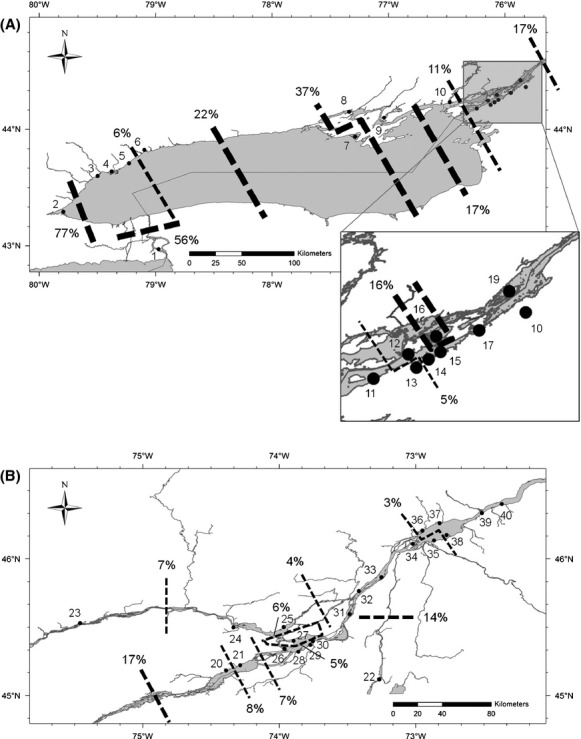
(A) First 20 barriers in Ontario sector and in (B) Quebec sector inferred by BARRIER. Lines thickness: barriers' relative strength, thick: >15%, medium: 5–15%, thin: <5%.

The spatial variation in patterns of IBD was also reflected by the geographic distribution of the first 20 barriers inferred by BARRIER (Fig.2). Thus, the majority of barriers were stronger in the Ontario (Fig.2A) than in the Quebec sector (Fig.2B), and the strength of barriers significantly decreased from upstream Lake Ontario to downstream St. Lawrence River (Spearman's *ρ* = −0.6963, *P *=* *0.0013). Accordingly, global *F*_ST_ values were three times higher in the Ontario sector than in Quebec sector considering annexes (*F*_ST_ = 0.0297, *P *<* *0.001 vs. *F*_ST_ = 0.0100, *P *<* *0.001, respectively) and excluding annexes (*F*_ST_ = 0.0186, *P *<* *0.001 vs. *F*_ST_ = 0.0051, *P *< 0.001, respectively).

Significant barriers determined the occurrence of 10 putative populations within the Ontario sector (Fig.2A) and 8 putative populations within the Quebec sector (Fig.2B, details table S8). The AMOVAs revealed that there was more variation between these groupings than between sites within them for the whole study area (2.03% vs. 0.22%, *P *<* *0.00001; Table1). There was stronger structuring between the 10 groupings within the Ontario sector (2.84%) than between the 8 groupings of Quebec sector (1.05%; Table1). Mean pairwise *F*_ST_ values between these grouping are reported at Table S7 for comparison.

**Table 1:** AMOVAs on population groupings.

Groups compared	Component	df	Sum of squares	% variation	*P*-value
Within whole study area (*N* = 18 groupings)	Among groups	17	882.074	2.03	<0.00001
	Among sites	22	197.839	0.22	<0.00001
	Within sites	5786	38991.899	97.74	<0.00001
Within Ontario sector (*N* = 10 groupings)	Among groups	9	452.568	2.84	<0.00001
	Among sites	9	86.320	0.40	<0.00001
	Within sites	2151	14310.747	96.64	<0.00001
Within Quebec sector (*N* = 8 groupings)	Among groups	7	263.836	1.05	<0.00001
	Among sites	13	111.519	0.15	<0.00001
	Within sites	3635	24681.152	98.80	<0.00001

### Landscape genetics

Among the 20 environmental variables, Mantel's tests identified six variables most correlated with pairwise *F*_ST_ (Mantel's *r*^2^* *>* *0.10 and *P *<* *0.05, in addition to waterway geographic distance; Table2). Multiple regression on distance matrices full model (Table3) revealed that pairwise data on dam index (*b *=* *0.4852, *P *=* *0.0029), spring water conductivity (*b *=* *0.3617, *P *=* *0.0001) and interannual water level stability (*b *=* *0.3499, *P *=* *0.0178) were significantly correlated with pairwise *F*_ST_ values (*R*^2^* *=* *0.6411, *P *=* *0.0001; Table3), whereas the other two variables were not significant. The final model comprising the three significant variables presented a similar level of explained variance (*R*^2^* *=* *0.6397, *P *=* *0.0001; Table3).

**Table 2:** Mantel tests results of pairwise *F*_ST_ matrix coupled with environmental variables matrices and selection of variables for subsequent multivariate analysis (*r *>* *0.10 and waterway distance). pH variable was discarded because of collinearity with spring water conductivity. For details on variables, see Table S4A.

Environmental variable	Mantel's *r*	*P*-value	Selection
Dam index	0.4155	0.0011	×
Interannual water level stability	0.2780	0.0031	×
Spring water conductivity	0.1573	0.0004	×
pH	0.1544	0.0113	
Spring water temperature	0.1534	0.0127	×
Hydrography	0.1231	0.0001	×
Current velocity	0.0923	0.0049	
Tide amplitude	0.0842	0.0196	
Wetlands fragmentation	0.0709	0.0037	
Waterway distance	0.0511	0.0123	×
Toxics contaminated areas	0.0458	0.0257	
Inorganic compounds contamination	0.0446	0.0473	
Fish BPC contamination	0.0243	0.0629	
Water masses	0.0180	0.0337	
Organic compounds contamination	0.0083	0.0492	
Sediment types	0.0073	0.3401	
Water color	0.0038	0.2584	
Navigation channel	0.0002	0.4076	
Fish mercury contamination	<0.0001	0.5265	
Turbidity	<0.0001	0.4565	

**Table 3:** Multiple regression on distance matrices (MRM) model on environmental variables with Pearson's correlation coefficient and pairwise *F*_ST_ matrix as a response variable. *b*: standard partial regression coefficient; *R*^2^: coefficient of multiple determination; *F*: Fisher's pseudo-test value.

Model components	Full model		Final model	
	*b*	*P*	*b*	*P*
Dam index	0.4852	0.0029	0.5015	0.0009
Spring water conductivity	0.3617	0.0001	0.3493	0.0001
Interannual water level stability	0.3499	0.0178	0.3375	0.0178
Hydrography	−0.0419	0.7113	–	–
Waterway distance	0.0329	0.7922	–	–
Spring water temperature	0.0198	0.8816	–	–
Model significance	*R*^2^	*P*	*R*^2^	*P*
	0.6411	0.0001	0.6397	0.0001
Fisher's test	*F*	*P*	*F*	*P*
	48.83	0.0001	98.85	0.0001

**Figure 3:**
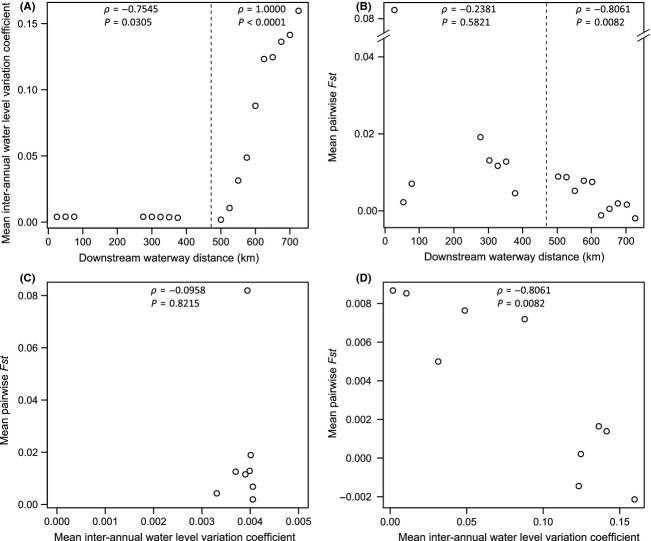
Sliding windows of sites located in a 50 km window that moves following water flow across system with a 25 km step. A) Mean inter-annual water level variation coefficients and B) mean pairwise *Fst* values between sites as a function of middle downstream waterway distance from the upstream farthest point (site 2) of each window. Mean pairwise *Fst* values between sites as a function of mean inter-annual water level variation coefficients for C) above and D) below Moses-Saunders regulation dam.

Downstream of the Moses-Saunders dam, water level variation was high and steeply increased with downstream waterway distance (*ρ* = 1.0000, *P *<* *0.0001; Fig.3A), contrary to upstream Moses-Saunders dam where water level variation was very low and the slope was less pronounced and the relationship much weaker (*ρ* = −0.7545, *P *=* *0.0305; Fig.3A). At the scale of the whole study area, the pattern of spatial genetic differentiation was extremely dissimilar within the Ontario and Quebec sectors. Mean pairwise *F*_ST_ values decreased significantly with downstream waterway distance below the Moses-Saunders dam (*ρ* = −0.8061, *P *=* *0.0082; Fig.3B), whereas this relationship was not significant above that dam (*ρ* = −0.2381, *P *=* *0.5821; Fig.3B). Upstream of the Moses-Saunders dam, mean pairwise *F*_ST_ values were not correlated to mean interannual water level variation coefficients (*ρ* = −0.0958, *P *=* *0.8215; Fig.3C), whereas a strong negative correlation was observed downstream of the dam (*ρ* = −0.8061, *P *=* *0.0082; Fig.3D).

## Discussion

Globally, northern pike presented weak but spatially variable genetic structure throughout the whole study area and from 20 environmental variables tested, three were found to significantly influence the observed pattern of population structure: interannual water level variation, different water masses and dam's presence.

### Landscape genetics

#### Interannual water level variation

**Figure 4:**
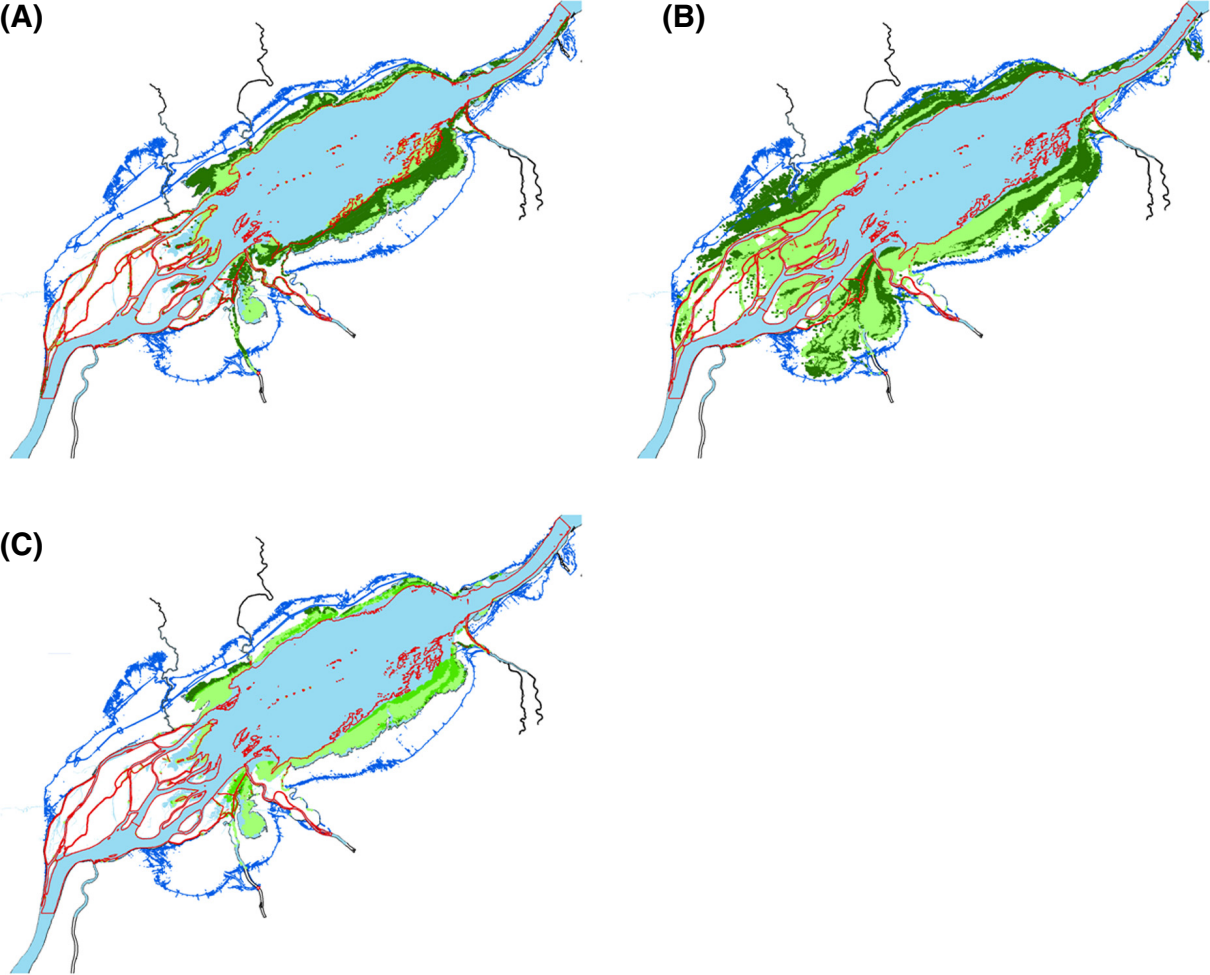
Modeled suitable spawning habitats for northern pike within Lake St. Pierre in lower SLR for two extreme water levels scenarios; (A) 1965: low level, (B) 1997: high level. (C) Overlap of (A) and (B) spawning habitat surfaces considering habitat quality. Blue/red lines: flood plain upper limit at maximum/minimum water levels, respectively. Green: suitable spawning habitats, from high (dark) to low quality (light). Data set originates from Mingelbier et al. (2008).

The Lake Ontario (LO) exhibits low water level variability mainly due to natural lake topography, while the St. Lawrence River (SLR), narrower and fed by many important tributaries, exhibits high water level variations. Downstream Moses-Saunders regulation dam (lower SLR, Quebec sector) we observed that interannual water level variation was negatively correlated to the extent of population genetic structure. Yearly localization and quality of spawning habitat were previously modeled as a function of annual water levels within lower SLR by Mingelbier et al. (2008). This revealed that interannual water level variations largely modulate localization, surface and access to spawning areas (Mingelbier et al. 2008). For example, in Lake St. Pierre within the lower SLR, modeled northern pike suitable spawning habitat spatially differed for two extreme water level scenarios (Fig.4; data from Mingelbier et al. 2008).

Such temporal habitat variability, in addition to habitat degradation and fragmentation from human activities, may impact the temporal stability and the extent of population structure, especially in areas that are more affected by water level variations. At a given locality, the more water levels vary interannually, the more suitable spawning habitats do not spatially coincide between years. It implies that depending on annual water level, available spawning habitat location and quality will vary from year to year. Consequently, adult northern pike ready to spawn may be unable to return to their previously suitable natal site which could be completely exundated during that year. In such a case, they would possibly seek an alternative suitable spawning area. This would hypothetically limit adult northern pike's ability to return to spawn at their natal site and, consequently, would impede the maintenance of a pronounced local population genetic structure. Genetic differentiation varied negatively as a function of interannual water level fluctuation that decreased from upstream to downstream, which may partly explain the weak population genetic structure in the Quebec sector (lower SLR). In the Ontario sector (LO and upper SLR), the much lower interannual water level variability may have favored the development of a stronger genetic structure because of a higher probability of homing to natal sites.

To our knowledge, only a handful of studies have investigated the impact of water level fluctuations in any aquatic species. Water level variation has previously been shown to influence the extent of gene flow and migration rates in brown trout (*Salmo trutta*, Ostergaard et al. 2003) whereby occasional but recurrent drought events were reported to cause frequent extinction/recolonization events, resulting in strong genetic drift, high gene flow and instability of genetic structure over time. In eastern mosquitofish (*Gambusia holbrooki*), when severe episodic drying events happens in the Everglades in Florida, individuals from different populations intermix in deep-water refuges during dry years where only patches of habitat are available (McElroy et al. 2011). During a wet year, population genetic structure presented a patchy pattern that was related to heterogeneity within regions while during a dry year, population genetic structure was very weak and a Walhund effect was detected. These episodic droughts were hypothesized to lead to reproduction between individuals belonging to different populations. This phenomenon of population's genetic homogenization by intermittent gene flow could equally concern the lower SLR where population genetic structure was very weak and water level variation very high. More generally, habitat variability has been related to the extent of population structure in other taxonomic groups as well. For instance, some species of aquatic insects that are specialists of unstable environments characterized by periodic drought events exhibits stronger dispersal which results in weaker genetic structure (Monaghan et al. 2005; Baggiano et al. 2011).

#### Environmental factors enhancing population structure

In addition to temporal variability, the LO–SLR system exhibits profound spatial heterogeneity, including the presence of different water masses. Differences in water conductivity between sampling sites, which is a reliable proxy of relative membership to different water masses (Désilets and Langlois 1989), were positively correlated with the extent of genetic differentiation in the Quebec sector. For example, the boundary separating the two groupings within Lake St. Louis [north shore (site 27) vs. south shore (sites 26-28-29-30), Fig.2B] spatially corresponds to the pronounced boundary of the very distinct waters masses from the LO and the Ottawa River (CSL 1996; Leclerc et al. 2008). Given that LO–SLR system's water masses have very different physicochemical properties (Table S1; CSL 1996), this could contribute to enhance populations divergence on either side of the mixing area of different water masses (as in Egea-Serrano et al. 2009). As the water masses flow in parallel through SLR with limited lateral mixing (CSL 1996), they also could physically limit passive dispersal of larvae and young fish (Roberts 1997). In freshwater ecosystems, the possible influence of physicochemically distinct water masses influence on population structure has been invoked only once (Leclerc et al. 2008), also in the SLR. In marine ecosystems, however, currents and water mass origin have often been shown to affect dispersal in fish (e.g., Logerwell et al. 2005; Pampoulie et al. 2006; White et al. 2010).

As reported previously for other species (Yamamoto et al. 2004; Heggenes and Røed 2006), and as generally expected, the occurrence of dams is also linked to genetic structure discontinuities, and the six dams located within the LO–SLR (Fig.1) coincide with putative boundaries of population units (Fig.2). These dams potentially impeded northern pike dispersal since their construction (1929, 1939 and 1979), although larvae and adults may still passively or actively disperse through in upstream/downstream direction. For instance, downstream dispersal of *Cottus gobio* was only lightly affected by dams (Junker et al. 2012), leading to asymmetrical gene flow.

Five of the six dams of the LO–SLR system mostly spatially coincide with historical natural obstacles at the landscape scale. For example, Moses-Saunders dam was built between Ontario and Quebec sectors where SLR rapids occurred historically. This suggests that natural barriers has been likely contributing for a longer period to shape the present northern pike population structure, which could have been reinforced by dams presence during the last century. Particularly, Moses-Saunders dam likely have increased the existing pattern of population genetic structure already created by SLR rapids between the upstream (Ontario sector) versus downstream (Quebec sector) part of our study area.

Northern pikes tolerate a wide range of environmental conditions, but they choose their spawning habitat according to several environmental factors, including vegetation type and density, current velocity, water temperature, water depth, turbidity, and dissolved oxygen concentration (Casselman and Lewis 1996; Mingelbier et al. 2008). Here, water depth, which is implied into the variable of interannual water level variation, is also implicitly related to habitat selection (see above). As a comparison with pike, a similar study was realized in lower SLR for yellow perch (*Perca flavescens*, Leclerc et al. 2008) that also uses flood plains for spawning during early spring. Likewise, dam's presence and water masses boundaries were involved in explaining yellow perch genetic structure in this area. However, interannual water level variation was not taken into account in that study. Boundaries of population units observed in both species almost perfectly spatially coincided (Leclerc et al. 2008). This suggests that taxonomically very distinct species from a same ecosystem that share characteristics of their reproductive ecology may be affected in the same manner by abiotic factors.

### Variable extent of population structure and comparisons with other studies

Weak overall population genetic structure was observed in our study area, but it was clearly much weaker in the lower SLR than in LO and upper SLR. We defined the occurrence of 10 populations in Ontario sector (LO and upper SLR) and eight populations in Quebec sector (lower SLR). Thus, because the extent of genetic variance between sample groupings defined by BARRIER was significant and because it was about 10 times more pronounced than between sampling sites within them, we consider these groupings as the most likely genetically distinct pike populations in the study area. Even if these groupings are weakly differentiated, we propose them as populations units as our results refuted panmixia and the extent and pattern of genetic structure of these populations varied significantly and non-randomly as a function of landscape features. It is also important to remember that strong genetic connectivity may nevertheless translate into relatively weak demographic connectivity (Waples and Gaggiotti 2006; Lowe and Allendorf 2010). Consequently, and despite the ongoing debate of what is a population and how does it pertain to usefulness for management (Waples and Gaggiotti 2006), it would seem warranted to consider that the demography of northern pike is regulated by a metapopulation dynamics (Hanski 2004) rather than assuming total panmixia. The extent of population genetic structure we documented was also lower than previously reported for northern pike in another large open system on a similar geographic scale. For example, northern pike's global genetic differentiation was notably higher over 800 km along the coastal Baltic Sea (*F*_ST_ = 0.032, Laikre et al. 2005), than for LO–SLR system (*F*_ST_ = 0.021), and much higher than populations of the Quebec sector (lower SLR) (*F*_ST_ = 0.005). Baltic Sea is an almost enclosed water body which does not experience major interannual water level variability as it is the case for the lower SLR, and this could partly explain the weaker genetic structure observed in this study. More generally, at a large geographic scale, low levels of population genetic structure have been reported for northern pike in connected habitats (e.g., Miller et al. 2001; Laikre et al. 2005) but as expected, more pronounced (yet moderate) levels were documented between disconnected habitats (e.g., reviewed by Miller and Senanan 2003), as we observed here between more geographically isolated sites (i.e., comparisons involving the four annexes). At a smaller scale, low but significant genetic differences have been reported between proximate sampling sites (Miller et al. 2001; Bosworth and Farrell 2006), akin to what we observed for the Ontario sector and some pairs of sites in Quebec region where interannual water level variation was minimal. Although northern pike has been reported to be sedentary (Bosworth and Farrell 2006; Vehanen et al. 2006), long-distance movements have been reported (e.g., 130 km over 3 days, Massé et al. 1988). This suggests that this species can substantially disperse over small and large geographic areas, which may contribute to the globally low level of genetic differentiation in comparison with most freshwater species (DeWoody and Avise 2000). IBD relationship showed that clinal genetic variation played a significant explanatory role in the observed genetic structure but more strongly in LO than in SLR. Also, slopes of the IBD relationships showed that geographic distance had a spatially variable impact on the extent of genetic connectivity. Thus, geographic distance apparently played a greater role in explaining the pattern of population genetic structure observed upstream than downstream of the study area.

### Implications for management

Admittedly, the extent of population structure and genetic connectivity we documented in this study can be qualified of weak at best. More generally, systems characterized by such weak population genetic structure are particularly challenging because of the potential limitations in inferences they cause by using the most commonly used statistical approaches. For instance, the STRUCTURE and DAPC analyses could not be much informative in our study except to confirm the presence of a very subtle pattern of population genetic structure, but without defining it in terms of characterizing evident and cohesive population clusters. In an applied context, it is hence a very delicate challenge to elaborate management plans for a system where the panmixia hypothesis is statically ruled out but where the population genetic structure is so weak that most conventional analyses are not powerful enough to clearly define the existing population structure. Nevertheless, it is essential to pay attention to the biological significance of such subtle patterns because it is well known from theory that large genetic connectivity resulting from long-term gene flow between populations may be associated with level of demographic connectivity that are consequential for management purposes (Waples and Gaggiotti 2006). Here, in view of the spatially variable extent of population structure, northern pike management perspectives should take into account the influence of interannual water level variation on population structure. Regions of lower SLR that are more prone to water level variation (Lake St. Louis and downstream) should be precautionary managed, given that northern pike population units from these areas, although genetically distinct, may be highly connected demographically (Lowe and Allendorf 2010) and hence local overfishing could impact northern pike stocks over a wide geographic area. Especially where the habitat is stable and the structure clearly apparent, (LO and upper SLR), northern pike should be managed as a metapopulation (Hanski 2004), which would predict a global impact of local exploitation given the partial demographic connectivity in such systems.

### Future research directions

This study opens a way to research avenues that should be followed for enhancing comprehension of LO–SLR system's dynamics. Given the weakness of the detected genetic signal and since the possibilities of high-throughput technologies improved substantially as we undertook the study a few years ago, it would be relevant to perform these types of analyses using a large number of SNPs (for instance using genotyping-by-sequencing, GBS) which would allow increasing the precision (i.e., reducing the variance) of estimations of genetic parameters for a better resolution of magnitude of genetic differentiation and connectivity (Allendorf et al. 2010). Furthermore, performing simulations to evaluate the historical influence of anthropic features should be considered as a further step of this research program in order to more firmly validate our interpretations. Finally, to our knowledge, this study remains one of the very few examples of the effect of spatial and short-term temporal habitat variability impeding the development of population genetic structure, thus highlighting the relevance of performing similar studies in other taxonomic groups.

## Supporting Information

**Table S1.**Physicochemical characteristics of water masses of lake Ontario and St. Lawrence River system between 1985 and 1990 (data from CSL 1996). **Table S2.** Sample statistics related to each sampling site and analyzed at 22 microsatellite loci. **Table S3.** Microsatellite loci characteristics. *N*_*A*_: Number of alleles per locus; *Ta*: PCR annealing temperature in degrees Celsius. **Table S4.** Environmental variables included in landscape genetics analyses. **Table S5.** Analyses of molecular variance (AMOVA) (A) between sampling years (B) between sampling periods (pre and during spawning only). **Table S6.** Multilocus pairwise *F*_ST_ values (Weir & Cockerham 1984) above diagonal and associated *p*-values below diagonal. **Table S7.** Mean pairwise *F*_ST_ values between population groupings in (A) Lake Ontario and (B) St. Lawrence River. **Table S8.** Definition of putative population groupings, from upstream to downstream.
